# Supplementary figures and images for: Ex vivo and in vivo CRISPR/Cas9 screenings identify the roles of protein N-glycosylation in regulating T-cell activation and functions
Source: eLife. 2026 Mar 20;14:RP108724. doi: 10.7554/eLife.108724 (PMC13004595; doi:10.7554/eLife.108724)

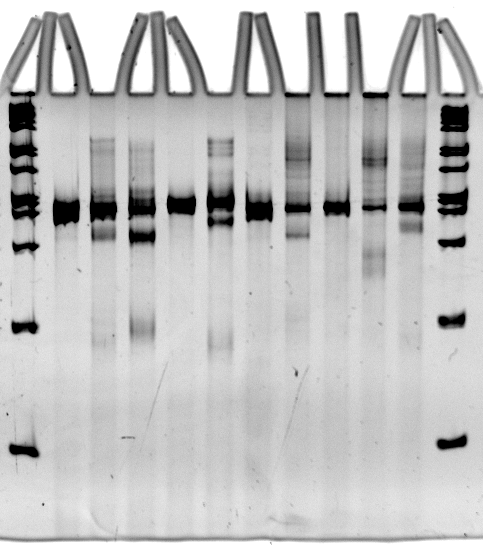

Supplement: Figure 1—figure supplement 2—source data 1. [file elife-108724-fig1-figsupp2-data1.zip › Figure 1–figure supplement 2-source data 1/Gel-1.tif]

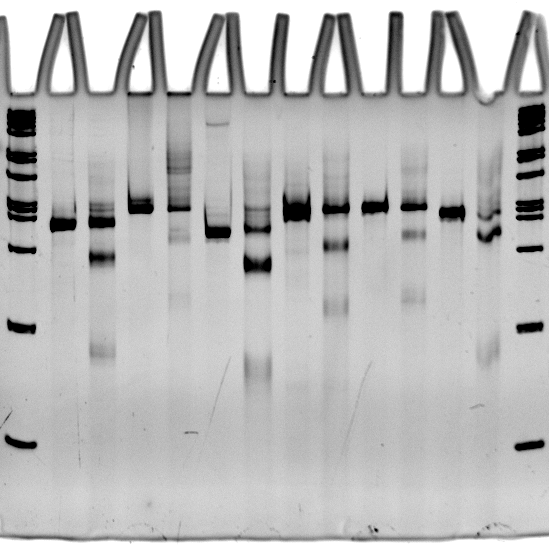

Supplement: Figure 1—figure supplement 2—source data 1. [file elife-108724-fig1-figsupp2-data1.zip › Figure 1–figure supplement 2-source data 1/Gel-2.tif]

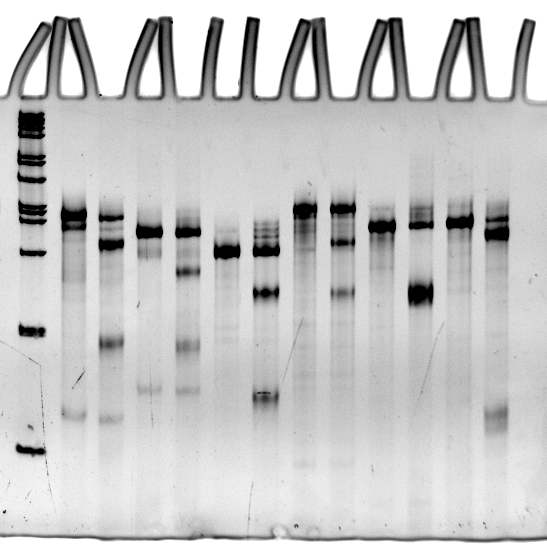

Supplement: Figure 1—figure supplement 2—source data 1. [file elife-108724-fig1-figsupp2-data1.zip › Figure 1–figure supplement 2-source data 1/Gel-3.tif]

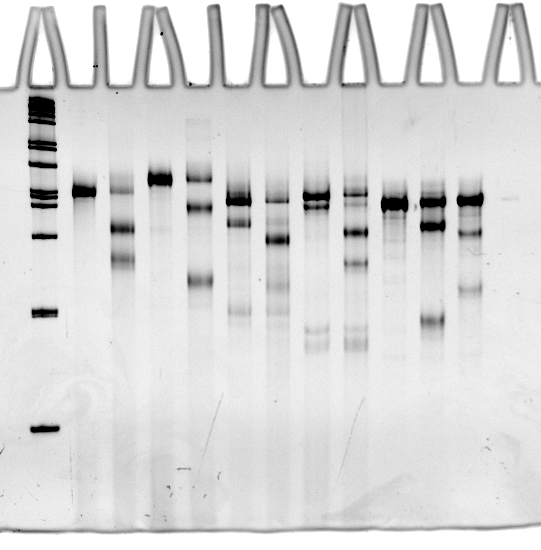

Supplement: Figure 1—figure supplement 2—source data 1. [file elife-108724-fig1-figsupp2-data1.zip › Figure 1–figure supplement 2-source data 1/Gel-4.tif]

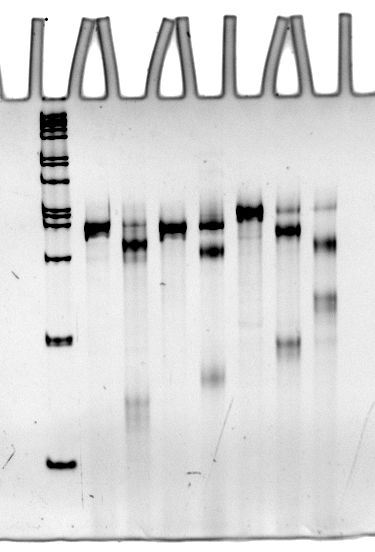

Supplement: Figure 1—figure supplement 2—source data 1. [file elife-108724-fig1-figsupp2-data1.zip › Figure 1–figure supplement 2-source data 1/Gel-5.tif]

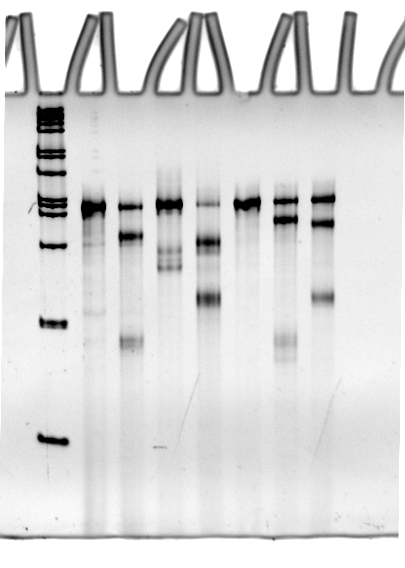

Supplement: Figure 1—figure supplement 2—source data 1. [file elife-108724-fig1-figsupp2-data1.zip › Figure 1–figure supplement 2-source data 1/Gel-6.tif]

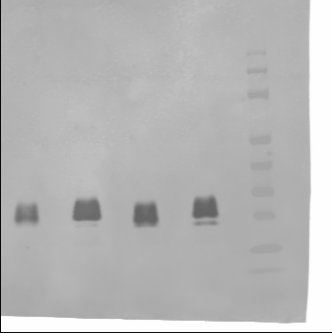

Supplement: Figure 5—source data 1. [file elife-108724-fig5-data1.zip › Figure 5d-e source data/Figure 5d original western blot of CD8β.jpg]

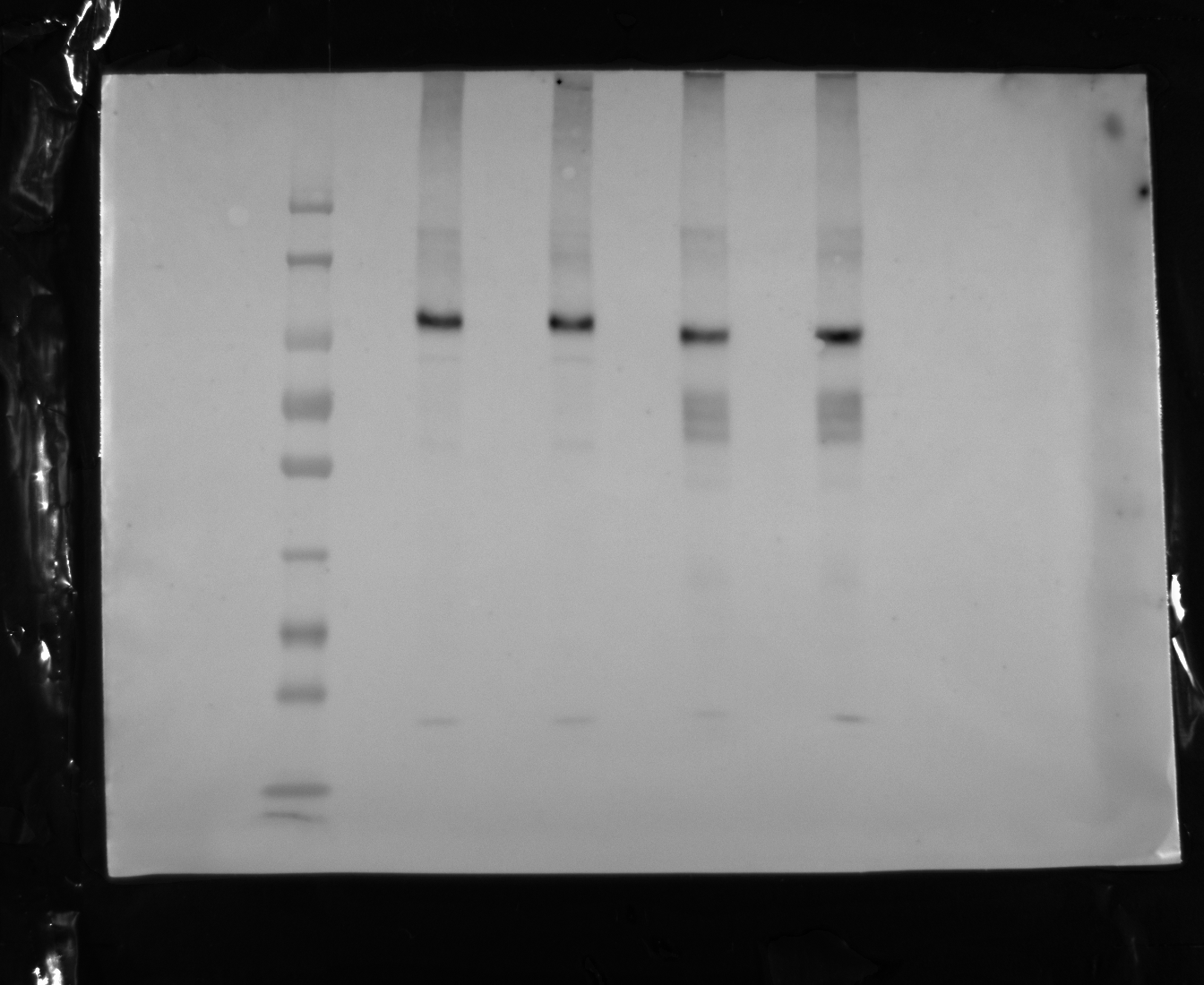

Supplement: Figure 5—source data 1. [file elife-108724-fig5-data1.zip › Figure 5d-e source data/Figure 5e original western blot of Itgb7.tif]

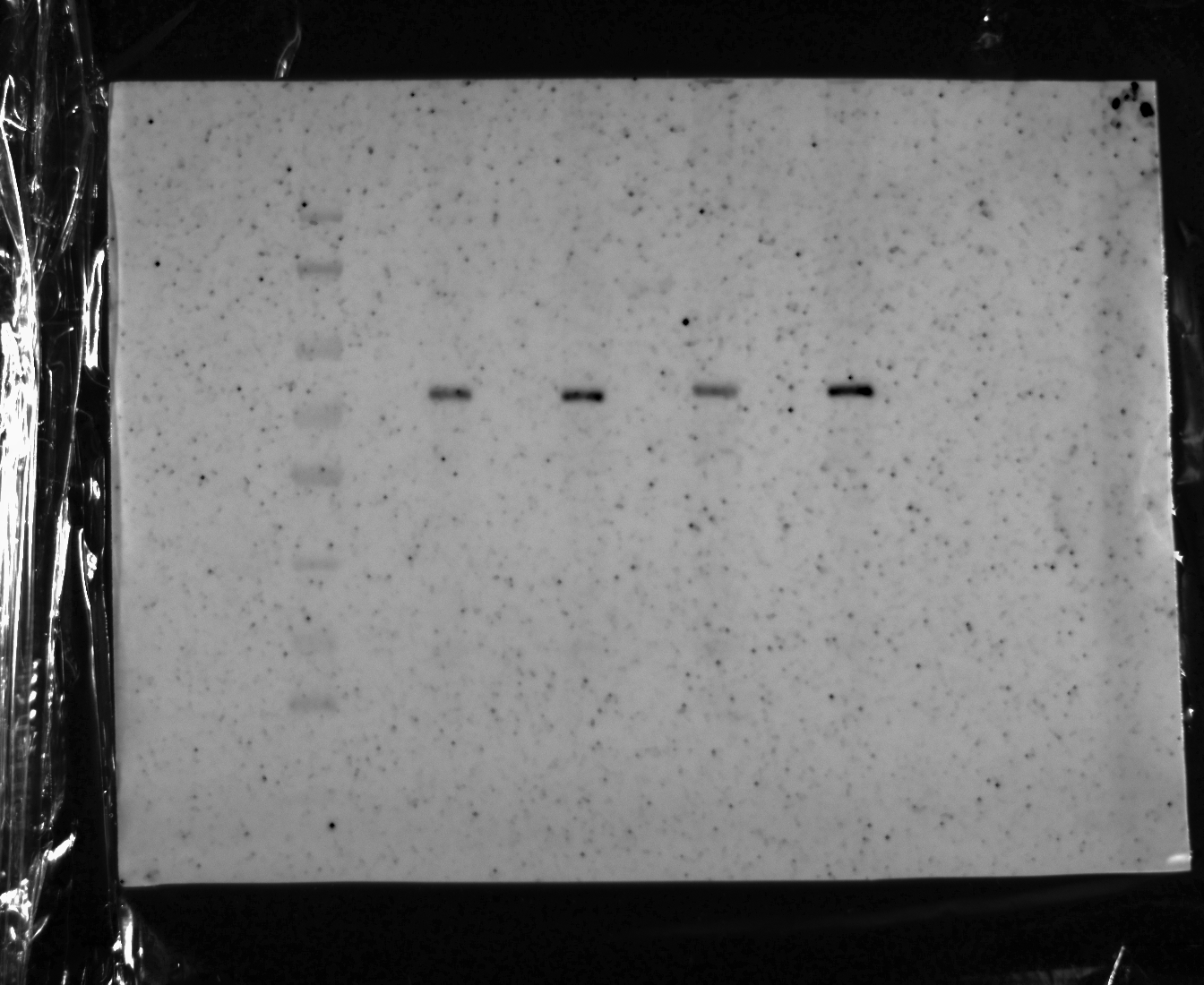

Supplement: Figure 5—source data 1. [file elife-108724-fig5-data1.zip › Figure 5d-e source data/Figure 5e original western blot of Sell.tif]
