# Supplementary material for: Ex vivo and in vivo CRISPR/Cas9 screenings identify the roles of protein N-glycosylation in regulating T-cell activation and functions
Source: eLife. 2026 Mar 20;14:RP108724. doi: 10.7554/eLife.108724 (PMC13004595; doi:10.7554/eLife.108724)
Supplement: Figure 1—figure supplement 2—source data 2. [file elife-108724-fig1-figsupp2-data2.zip › Figure 1¿Cfigure supplement 2-source data 2.pdf]

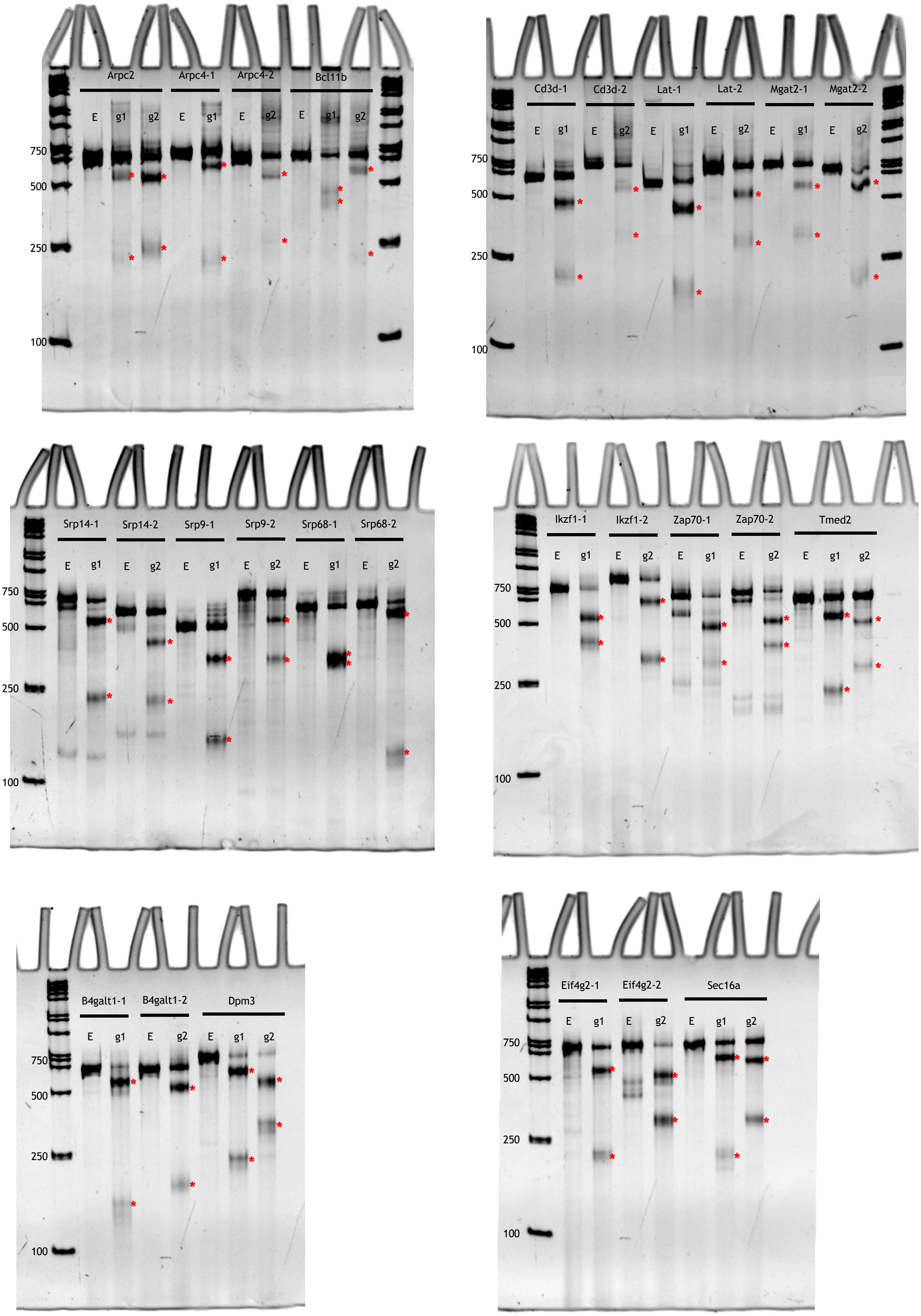

Figure 1-figure supplement 2-source data 2

PDF file containing original PAGE gel for Figure 1–figure supplement 2, indicating the genes, gRNAs and relevant bands.
