# Supplementary material for: Ex vivo and in vivo CRISPR/Cas9 screenings identify the roles of protein N-glycosylation in regulating T-cell activation and functions
Source: eLife. 2026 Mar 20;14:RP108724. doi: 10.7554/eLife.108724 (PMC13004595; doi:10.7554/eLife.108724)
Supplement: Figure 5—source data 2. [file elife-108724-fig5-data2.zip › Figure 5-source data 2.pdf]

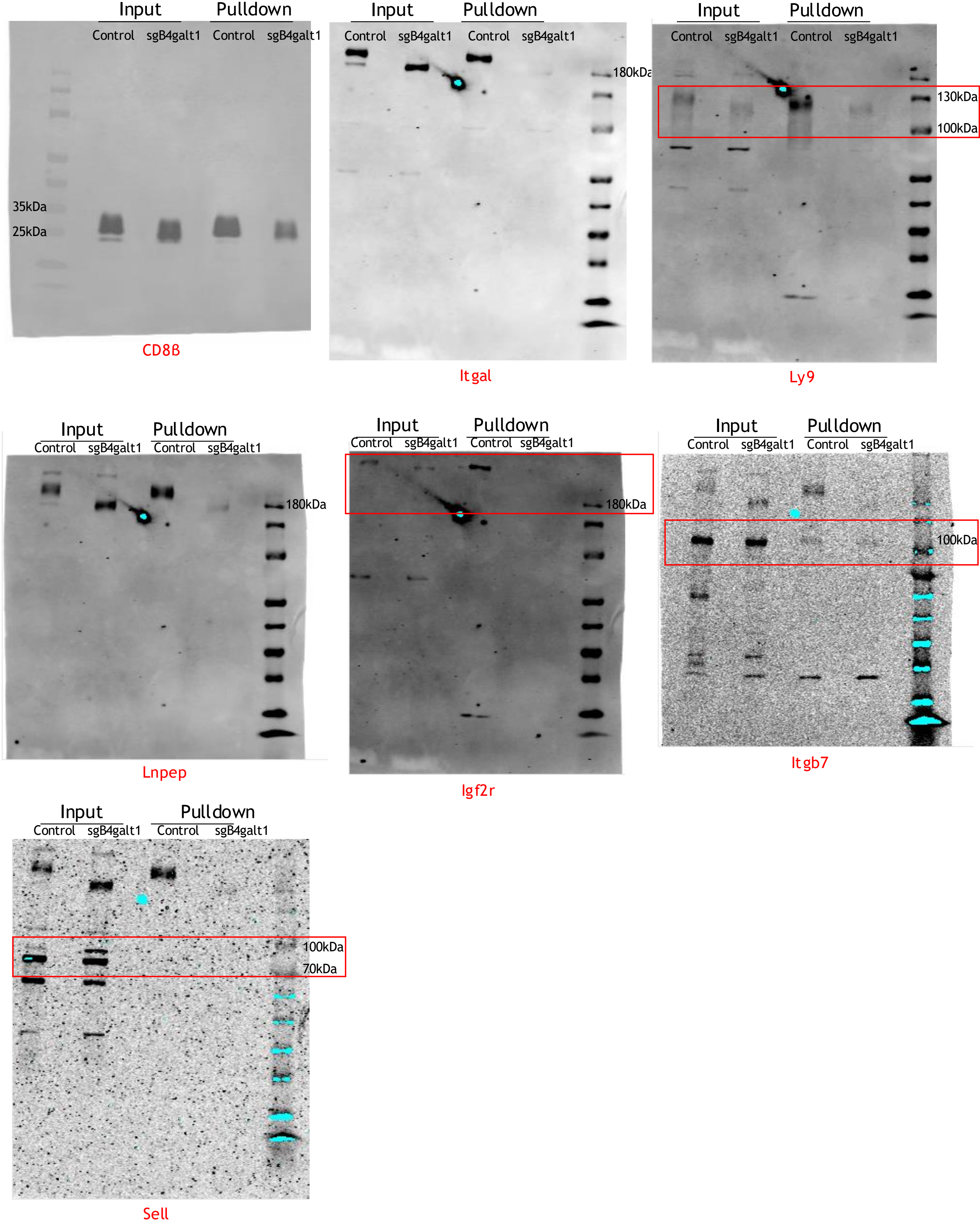

Figure 5d-source data

PDF file containing original western blots for Figure 5d, indicating the relevant bands and treatments.

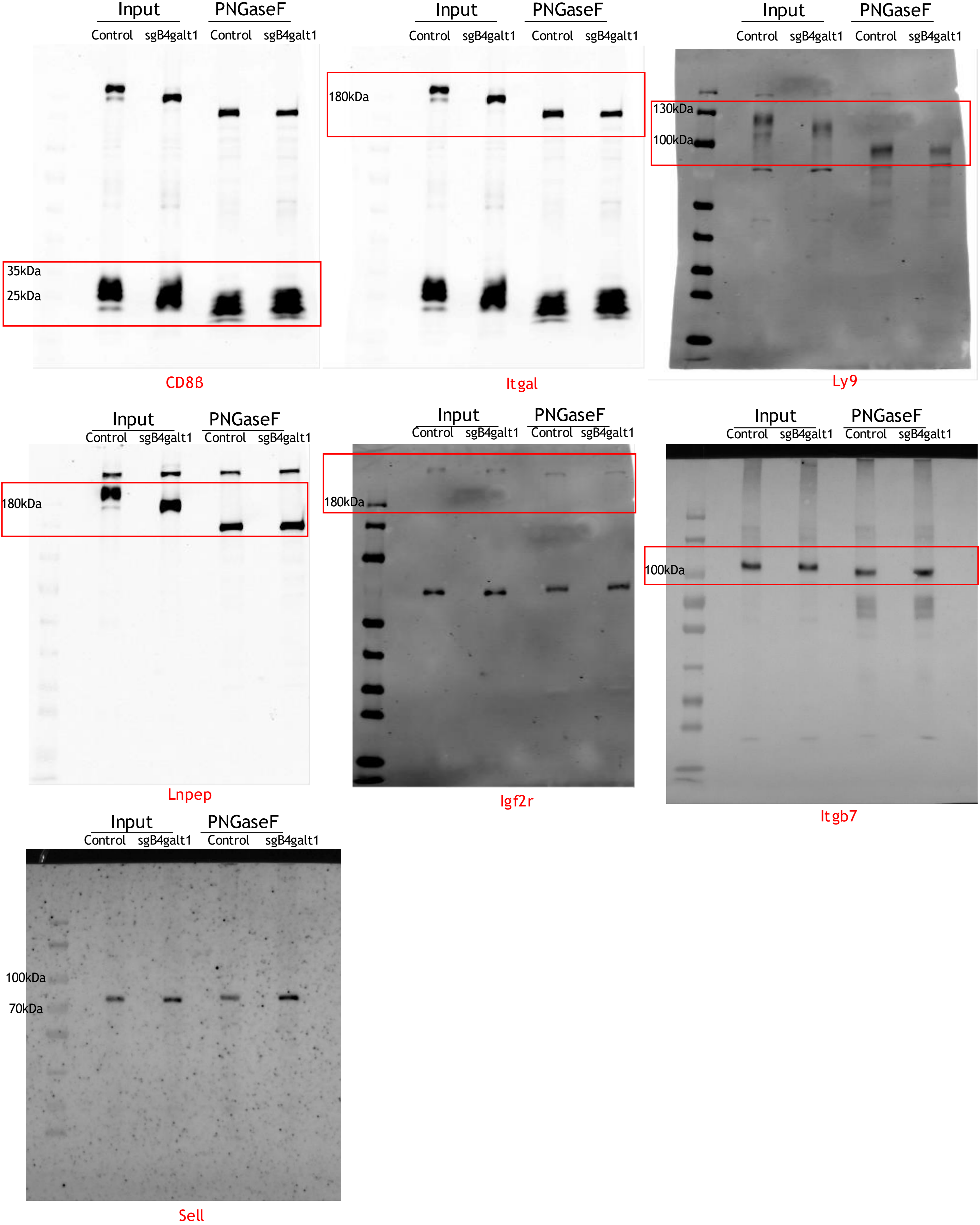

Figure 5e-source data

PDF file containing original western blots for Figure 5e, indicating the relevant bands and treatments.
